# Animal Modeling of Pediatric Liver Cancer

**DOI:** 10.3390/cancers12020273

**Published:** 2020-01-22

**Authors:** Richard S. Whitlock, Tianyou Yang, Sanjeev A. Vasudevan, Sarah E. Woodfield

**Affiliations:** 1 Divisions of Pediatric Surgery and Surgical Research, Michael E. DeBakey Department of Surgery, Pediatric Surgical Oncology Laboratory, Texas Children’s Surgical Oncology Program, Texas Children’s Liver Tumor Program, Dan L. Duncan Cancer Center, Baylor College of Medicine, Houston, TX 77030, USA; richard.whitlock@bcm.edu (R.S.W.); sanjeevv@bcm.edu (S.A.V.); 2 Department of Pediatric Surgery, Guangzhou Women and Children’s Medical Center, Guangzhou Medical University, Guangzhou 510623, Guangdong, China

**Keywords:** hepatoblastoma, children, mouse model, xenograft, patient-derived xenograft

## Abstract

Hepatoblastoma (HB) is the most common pediatric liver malignancy. Management of HB requires multidisciplinary efforts. The 5-year overall survival of this disease is about 80% in developed countries. Despite advances in the care of these patients, survival in recurrent or treatment-refractory disease is lower than 50%. This is due to more complex tumor biology, including hepatocellular carcinoma (HCC)-like mutations and expression of aggressive gene signatures leading to chemoresistance, vascular invasion, and metastatic spread. The current treatment protocols for pediatric liver cancer do not incorporate targeted therapies, and the ability to test these therapies is limited due to the inaccessibility of cell lines and mouse models. In this review, we discuss the current status of preclinical animal modeling in pediatric liver cancer, primarily HB. Although HB is a rare cancer, the research community has worked together to develop a range of interesting and relevant mouse models for diverse preclinical studies.

## 1. Introduction

Hepatocellular malignancies are now a leading cause of cancer-related mortality in people of all ages, and children are affected by both hepatoblastoma (HB) and hepatocellular carcinoma (HCC). Notably, HB is the most common primary liver malignancy in children with an incidence of approximately 1.3 to 1.5 cases per million [1,2]. This disease is generally seen in patients younger than 5 years of age and is associated with prematurity, low birth weight, and non-chromosomal congenital defects [3,4,5,6]. Interestingly, more recent data has shown that the worldwide incidence of HB is increasing at a rate faster than all other pediatric malignancies [7]. The 3-year survival rate for cases of standard risk HB is 91% [8] while high-risk cases, defined as tumor growth in all hepatic segments (pretreatment extent of disease (PRETEXT) 4), presence of vascular invasion, or metastatic disease, have a 3-year survival rate of only 65% [9]. High-risk HB requires more intensive chemotherapeutic regimens, including high dose cisplatin, doxorubicin, vincristine, and irinotecan, and these non-targeted chemotherapies are associated with serious complications including deafness, renal toxicity, cardiotoxicity, and neutropenia related morbidity and mortality [10,11,12]. The aggressive nature of high-risk HB concomitant with the relatively low overall disease incidence has historically prevented substantial study of more effective chemotherapy regimens and targeted therapies due to the lack of available samples. Only three cell lines, HepG2, Huh-6, and HepT1, are widely available for laboratory studies [13,14,15,16]. Relevant animal models have been developed more recently as a response to this historical difficulty, allowing researchers to accurately recapitulate human tumors in murine models to further study tumor biology and new treatment algorithms in an effort to increase overall survival of patients with high-risk disease. In this review, we will discuss the historical course of animal modeling of HB and pediatric liver cancers with key models summarized in Table 1.

## 2. Models Generated with Subcutaneous Injection of Widely Available and Patient-Derived Cell Lines

The earliest work to generate animal models of HB involved the subcutaneous injection of fresh patient-derived cells [20,21,22]. As early as the 1980s, Hata and colleagues established an α-fetoprotein (AFP)-secreting model of HB using a patient sample termed HB-3 implanted into the subcutaneous space in the back of female nude (Balb/c nu/nu) mice [21]. The foundation for this research came from work with HCC mouse models that showed that subcutaneous implantation of cells led to generation of tumors in animals while cells injected directly into the liver showed limited growth [29]. The development of patient-derived subcutaneous xenografts in immunodeficient mice was then explored further in several papers [20,22]. In 1995, Desdouets and colleagues described the implantation of a pure epithelial HB patient sample into the subcutaneous tissues of athymic nude mice to establish a model to study proliferation and differentiation of HB [22]. Interestingly, neither the model nor the transplanted sample was shown to secrete AFP [22]. The following year, Fuchs and colleagues described successful transplantation of HB into athymic nude mice with a take-rate of 80% from patient-derived tumor cell suspensions injected into subcutaneous tissues [20]. In this work, xenograft tumors were generated from three non-pretreated samples and one sample that had undergone three cycles of chemotherapy [20]. All samples within this study represented fetal and embryonal histology [20].

Additional work then focused on use of the widely available HepG2, Huh-6, and HepT1 cell lines to grow subcutaneous tumors in immunocompromised mice [15,17]. First, in 1996, Pietsch and colleagues reported the growth of HepT1-derived tumors with subcutaneous injection of the HepT1 cell line [15]. In 2006, Schnater and colleagues subcutaneously injected HepT1, Huh-6, and HepG2 cells into the left flank of athymic nude (NMRI nu/nu) mice [17]. They reported that HepT1 did not grow in vivo after injection into subcutaneous tissues while Huh-6 and HepG2 cells had implantation rates of 70% and 50%, respectively [17]. Although the primary tumors in these models showed characteristics of HB, including histological and pathological similarities, a deficit of these models was a lack of extrahepatic disease or distant metastases.

In general, subcutaneous models of various cancers have multiple advantages, including ease of access in measuring tumor size and in monitoring experimental treatment effects. Subcutaneous models are deficient in their ability to recapitulate tumor microenvironments, robust vascularization, and metastasis, however.

## 3. Models Generated with Splenic Injection of Widely Available Cell Lines

In an attempt to grow HB tumors orthotopically in the livers of animals, investigators then explored the use of a splenic injection technique [17]. In a groundbreaking paper by Schnater and colleagues in 2006, before which no intrahepatic models had been published, HepT1, HepG2, and Huh-6 cell lines were injected into the spleens of athymic nude (NMRI nu/nu) mice in an effort to develop intrahepatic models of HB [17]. This work was based on the hypothesis that HB tumor cells could populate the liver parenchyma through “overflow”, initially by spillover and invasion into the splenic venous circulation, therefore spreading via the portal vein into the liver. Growth of tumors was monitored by measurement of AFP levels. In a discussion of the study results, the group reported that HepT1 did not grow intrahepatically after splenic injection and also that a greater number of HepG2 and Huh-6 cells were required to initiate tumor growth by splenic injection than by subcutaneous injection [17]. At the time of their analysis, the researchers reported that only Huh-6 cells were able to settle within the liver and this was through metastatic spread [17]. At the same time, this spread was at the expense of partial loss of differentiation features of the malignancy [17]. At the time of publishing this study, a clear positive of this model was the development of the first known intrahepatic HB model, allowing for more accurate study of tumor characteristics within a living animal with an intact tumor microenvironment. However, despite the advances of this technique, it was shown that the Huh-6 hepatic nodules differed from primary HB tumors and that the take rate for tumors was relatively low [17].

In a second publication by Ellerkamp et al. in 2011, the authors developed an intrahepatic model by injecting Huh-6 and HepT1 cells into the spleens of immunocompromised NOD.Cg-Prkdcscid IL2rgtmWjl/Sz (NSG) mice and performing splenectomy on the animals 2 min after injection with a high-temperature battery-cautery to block intrasplenic growth and promote intrahepatic tumorigenesis [8]. With splenectomy, 83% of Huh-6 injected animals and 50% of HepT1 injected animals developed tumors in the liver [8]. These intrahepatic tumors secreted AFP and showed positive immunostaining for AFP, EpCam, β-catenin, and E-cadherin [8]. The tumors generally presented in animals as small, multifocal nodules without a dominant mass in the liver. This is a difficult model to use for preclinical studies since it is challenging to quantify the tumor burden of multifocal nodules after treatment.

## 4. Intrahepatic Cell Line-Derived Xenograft Models

The next frontier for HB modeling was the development of intrahepatic xenografts grown from the widely available HepG2, Huh-6, and HepT1 cell lines directly injected into the livers of immunocompromised animals. While the intrasplenic and subcutaneous injection methods resulted in growth of tumors in animals from HB cell lines, the tumors that developed were small and multifocal, which made it challenging to quantify tumor burden. In addition, because of limitations in tissue availability, there remained a need to be able to grow consistent intrahepatic tumors that accurately displayed patient- and tumor-specific phenotypes from cell lines and not just from fresh patient tissues. Initially, in 2009, Ong et al. generated an intrahepatic xenograft model with injection of HepG2 cells into the portal vein of severe combined immunodeficiency (SCID) animals, but this model was only characterized with positron emission tomography (PET) scanning, computed tomography (CT) scanning, and immunohistochemistry for proliferation and markers specific to the drug treatments tested in the paper [18].

**Figure 1:**
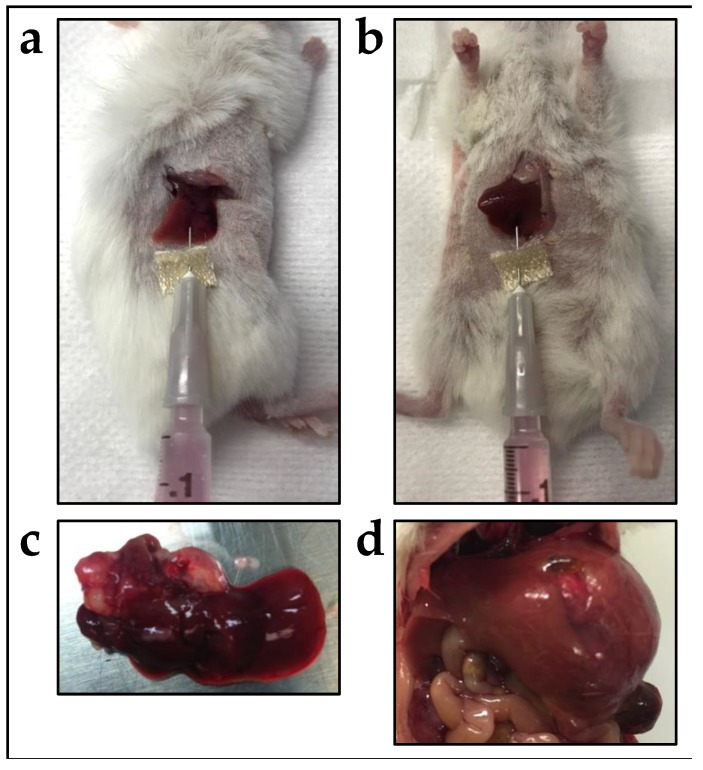
Injection of HepG2 and Huh-6 HB cells into the mouse liver to generate xenograft tumors. (**a**,**b**) Cells were injected either into the right median lobe (**a**) or the left lateral lobe (**b**). (**c**,**d**) Representative gross tumors generated with injection of Huh-6 cells into the right median lobe (**c**) or HepG2 cells into the left lateral lobe (**d**). From Woodfield et al., 2017 [19].

In 2017, Woodfield and colleagues developed several models with the growth of intrahepatic HB tumors from the commercially available HepG2 and Huh-6 cell lines [19]. In this work, cell lines were injected directly into either the right-median or left-lateral liver lobes of NOD/Shi-scid/IL-2Rγnull (NOG) mice [19]. Cells injected with both techniques grew large, extraphytic masses with local invasion indicative of PRETEXT 1, 2, and 3 disease. Figure 1 demonstrates this technique of intrahepatic injection of human tumor cells that was used in this study, with the resulting tumors shown. These intrahepatic HB xenograft models recapitulated the key hallmarks of the disease including elevated serum AFP levels, large exophytic tumors with active blood supplies, and embryonal histological phenotypes with elevation of AFP, Glypican-3 (GPC3), and β-catenin proteins [19]. These models, especially the HepG2 model, also displayed a consistent gene expression profile similar to human HB tumors [19]. Noted in the study was the observation that gene expression of the Huh-6 cells was changed by growth in the murine liver and that this correlated with differences in histology [19]. However, even with these established limitations, these novel models utilizing HepG2 and Huh-6 cells will be usable for studies of new pre-clinical therapies for HB. In our experience with extensive preclinical drug testing with intrahepatic models, these models are much better suited for relevant drug testing because they show toxicities associated with diminished hepatic clearance in the presence of biliary obstruction from a mass effect of tumor within the liver. It is well established that many chemotherapies are metabolized by the liver and, thus, hepatic dysfunction can interfere with drug clearance [30,31]. In our work, we have seen drugs show toxicities in our intrahepatic tumor models that have not otherwise shown toxicities in testing in multiple other cancer models with tumors outside of the liver. These intrahepatic models better represent this situation that is very real for cancer patients, facilitating more meaningful drug testing. Importantly, this work also showed the use of bioluminescent imaging (BLI) with cell lines that are transduced with luciferase [19]. With intraperitoneal injection of luciferin, therefore, the cells emit a signal that can be measured to monitor growth. Imaging of tumors growing within living animals is otherwise mainly limited to magnetic resonance imaging (MRI) and ultrasound.

## 5. Patient-Derived Xenograft Models

As discussed above, the earliest work to generate animal models of HB involved subcutaneous injection of fresh patient-derived cells, and patient-derived xenograft (PDX) models for pediatric liver cancer had been discussed as early as the 1980s and 1990s [20,21,22]. The clear benefit of establishing PDX models is the ability to study tumors in animals that most accurately recapitulate human disease without the intermediate phase of in vitro growth that is thought to change cells. These models are especially needed for meaningful testing of novel anti-cancer treatments. With other tumors, scientists had been able to perform high throughput drug screening to establish predictors of clinical trial drug response with PDX models. For example, Gao and colleagues established approximately 1000 PDX models with diverse malignancies and tested 62 treatments across six indications [32]. With this work, the investigators were able to establish a system for the prediction of patient responses to clinical trial compounds.

A hallmark study in the development of PDX models for pediatric liver cancer came from Nicolle and colleagues in 2016 [23]. In this study, they described the establishment of 24 PDX models from 20 HB samples, 1 HCC sample, 1 transitional cell tumor, and 2 malignant rhabdoid tumors (MRTs) [23]. The fresh patient samples were implanted into the brown fat within the interscapular space of athymic nude animals [23]. With this work, they showed that cytogenetic markers and phenotypic features were similar to patient tumors [23]. It was also noted that rapid tumor growth, AFP levels, and gain of chromosome 20 were all indicators for chemotherapy resistance and poorer prognosis [23]. Interestingly, it was shown in this study that the ability to generate a PDX from a patient tumor sample was predictive of a poorer prognosis for the patient [23].

**Figure 2:**
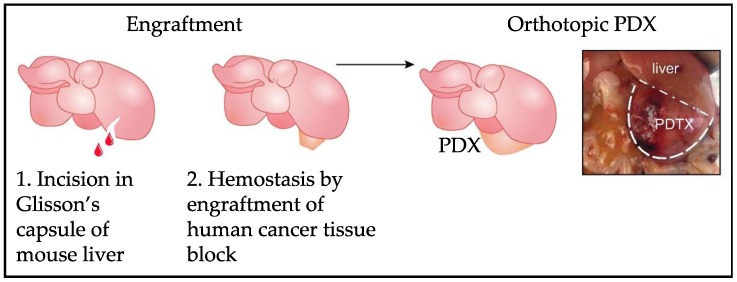
Implantation of whole tumor pieces onto the Glisson’s capsule of the mouse liver for generation of intrahepatic PDX HB tumors. Modified from Bissig-Choisat et al. (2016) [24].

The next forward push in this field of HB modeling came in the form of the development of intrahepatic PDX models. For multiple tumor types, previous work established that direct implantation of patient samples into organs of origin in animals led to the generation of models that most closely resembled primary disease. Up until this point, the majority of modeling research in the HB field had been performed with the subcutaneous and splenic injection techniques and with established cell lines, and these models were obviously limited. Bissig-Choisat and colleagues shared their experience with the creation of novel PDX models with the use of a new murine surgical implantation technique in 2016 [24]. While animals were under anesthesia, a 2 mm incision was made into the Glisson’s capsule of the mouse liver and a viable piece of primary patient tumor was engrafted upon the exposed liver parenchyma and sealed in place using a tissue adhesive (Vetbond) [24]. Figure 2 demonstrates this method of implanting human tumor samples directly onto an incision in the Glisson’s capsule of the murine liver. It was noted that these PDX models recapitulated the histologic, genetic, and biological characteristics of the primary tumors [24]. Importantly, these new PDX models also captured the metastatic behavior of the disease with reported metastasis in three PDXs observed as early as 9 weeks after implantation [24]. In this paper, these models were also shown to be usable for drug testing in that the authors showed growth inhibition with two therapies that targeted mutations found in the patient samples and models [24]. Therefore, this work unequivocally showed that these models recapitulated human disease and could be used to identify and validate individual therapeutic weaknesses [24]. Such work, however, is only possible at a high volume liver surgery center in order to meet the supply needs of necessary patient samples for the development of new PDX models.

## 6. Genetically Engineered Mouse Models

More recent work has focused on the development of genetically engineered mouse (GEM) models of the disease, including transgenic and knock-in/knock-out animals, with the aim of understanding how specific genetic aberrations contribute to disease initiation and progression. Although this work has shed much light on the pathogenesis of HB, generating GEM models tends to be costly and time-consuming. In addition, manipulation of genes in the germline can result in developmental problems or even lethality, preventing the desired studies from being completed and requiring the use of inducible or tissue-specific systems. A positive of using GEM models is that most models utilize immunocompetent animals, allowing relevant studies of the immune system in relation to the tumor and tumor microenvironment.

In 2014, Nguyen and colleagues established a GEM model with liver-specific overexpression of the oncofetal RNA-binding protein Lin28b, which is commonly increased in poorly differentiated and aggressive malignancies [25]. Prior to this, Lin28b and its role in tumor growth had not been adequately investigated. With this model, the investigators demonstrated that *LIN28B* overexpression was sufficient to induce both HB and HCC tumors in animals [25]. It was also reported that Lin28B was aberrantly activated in mouse models of Myc-driven HB, showing a potential tumorigenic role for Lin28b in combination with Myc [25]. Finally, knock-down or conditional silencing of *LIN28B* in these Myc-driven models was associated with reduced tumor burden and prolonged survival of animal models, supporting a role of Lin28B in tumor maintenance [25].

In a key paper in 2016, Comerford and colleagues reported the development of a model with perinatal coexpression of *myc* and mutant *CTNNB1* (the gene that codes for the β-catenin protein) within the developing mouse liver [26]. In this model, coexpression of both genes resulted in tumor development preferentially with HB over other liver tumor types in the neonatal mice [26]. Importantly, it was reported that these tumor models closely resembled human tumor tissues [26]. The idea for these models was interestingly derived from work with HCC models in which *myc* had been studied at a genetic level [33,34]. This work also showed clearly that *NFE2L2* functions as an oncogene in HB [26]. A major benefit of the GEM model that was established in this study was the identification of a potential genetic target that could be directly tested with treatment responses of potential therapies monitored in animals [26]. This was not a fully penetrant model, however, with fewer than half of the analyzed animals developing tumors, and many animals did not survive long enough after birth to be used in further studies [26].

In a second key paper in 2016, Zhu and colleagues extensively mapped the potential for tumors to form in major organs with the overexpression of various oncogenes through a lineage tracing Cre-recombination system driven from the *Prom1* locus that is expressed in a variety of cell types [27]. This work showed that liver tumors, including HB and HCC, originated with disruption of major cancer signaling pathways with conditional alleles, including *Kras^G12D^*, *ROSA^NICD1^*, *Pten^flx/flx^*, *Tp53^flx/flx^*, and *Cdkn2a^flx/fl^* [27]. This work also showed that only neonatal liver cells are tumorigenic but that damaging adult livers reactivates this “neonatal-like” tumorigenic behavior [27]. Although this work did not lead to the generation of a liver-specific GEM model, this research importantly contributed to an understanding of how liver cancer develops with aberrations in development and, in particular, provided a mechanism of how liver damage contributes to tumorigenesis in adults.

In addition, there are multiple GEM models that show a predisposition to develop liver cancer, particularly HCC. Many of these are liver repopulation models in which transplanted cells with a survival and growth advantage expand at the expense of the endogenous hepatocytes to repopulate the entire organ [35]. An interesting point about these models is that they are associated with an increased risk of liver cancer tumorigenesis [35]. The first of such models developed was the *Albumin-urokinase-plasminogen activator* (*uPA*) transgenic mouse, generated by Sandgren and colleagues in 1991 [36]. In these animals, expression of uPA in the liver causes hepatocyte toxicity, but rare hepatocytes that do not express the transgene can survive and repopulate the entire organ [36]. This model was further utilized in 1994 to show that adult normal mouse hepatocytes can be transplanted into uPA transgenic mice and will repopulate up to 80% of the endogenous, diseased liver [37]. Interestingly, it was observed as early as 1992 that hepatic tumor nodules also spontaneously develop in these animals, and these tumors resembled liver adenomas and HCCs [38]. A similar model developed in 1996 by Overturf and colleagues was the *Fumaryl-acetoacetate hydrolase* (*Fah*)-null model in which animals lack the Fah enzyme and, thus, accumulate fumaryl-acetoacetate and maleyl-acetoacetate [39]. This model represents hereditary tyrosinemia type I, in which accumulation of these compounds lead to progressive liver failure and early onset HCC [39,40]. Normal hepatocytes can be transplanted into these animals to repopulate most of the organ [39], or animals can be rescued by dosing with 2-(2-nitro-4-trifluoromethylbenzoyl)-1,3-cyclohexanedione or transfection with an adenoviral vector carrying the *Fah* gene [41,42]. This model, in particular, may be applicable to studies of pediatric liver cancer as children with hereditary tyrosinemia type I tend to develop liver cancer at a young age [43]. A third relevant mouse model that replicates a hereditary disease is a model generated with expression of the PiZ variant of human *Alpha 1-antitrypsin* in the livers of mice [44]. Like humans with alpha-1 antitrypsin deficiency (AATD), these mice accumulate the mutant protein in their livers and develop liver necrosis and inflammation [44]. In addition, older PiZ mice develop malignant liver tumors, mainly HCC and angiosarcoma, and hyperplastic nodules [45].

## 7. Models Generated with Hydrodynamic Tail Vein Injection with the Sleeping Beauty Transposon System

Additional work in the field of modeling pediatric liver cancer focused on the use of an innovative hydrodynamic tail vein injection technique to generate intrahepatic tumors with manipulation of liver tumor cells within animals, a technique developed by Liu et al. [46]. Briefly, injection of nucleic acids through the tail vein of animals in a large volume (10% of body weight) at a rapid rate (5 to 9 s) leads to the deposition of these nucleic acids primarily in the liver parenchyma cells because of specific anatomical features [47]. This is thought to result in transfection of 10% to 40% of hepatocytes [47]. Without stable somatic integration, these transfected genes are rapidly degraded. Therefore, this tail vein injection technique is combined with Sleeping Beauty transposase-mediated somatic integration for specific transfection of murine liver cells and stable, long-term target gene expression [48]. Interestingly, a fairly low percentage of hepatocytes are affected with this system, approximately 2% to 10%, which are surrounded by normal liver cells [47], and this sporadic transformation of a few hepatocytes resembles what is thought to occur with human disease. This model also avoids the costly and time-consuming breeding required for the generation of GEM models without sacrificing studies of genetic changes that contribute to oncogenesis. In addition, immunocompetent animals can be used with this modeling system. Finally, this system easily allows the overexpression or knock-down of multiple genes of interest that may work together in HB tumor initiation and progression. This technique was harnessed to manipulate liver cells to introduce cancer-associated mutations and overexpress oncogenes to measure their contribution to oncogenesis in a series of key publications.

The first study built on previous work that showed that most HB samples expressed both β-Catenin and Yes-associated-protein-1 (YAP-1) proteins and that coactivation of both was present in greater than 80% of samples [49]. Tao and colleagues showed in 2014 that dual hydrodynamic injection of both mutant, constitutively active *CTNNB1* and *Yap-1* led to hepatoblastogenesis in mice [28]. This study further demonstrated regulation of *Yap-1* levels by β-Catenin at the transcriptional level, and the in vivo findings strongly showed the synergistic effect that mutant *CTNNB1* and *Yap-1* dual activation had on HB tumor growth [28]. With this specific model, most animals developed multifocal nodules that eventually encompassed the entire murine liver with tumor, with death of all animals by 11 weeks [28,47].

In a second key study from Wang and colleagues in 2016, the contribution of Myc to HB tumorigenesis was explored with a combination of the *CTNNB1*/*Yap-1* hydrodynamic tail vein injection/Sleeping Beauty transposon model with knock-out of the *myc* locus [50]. This work showed that *myc* is not required for in vivo HB tumor initiation but is necessary for sustained tumor growth within animals [50]. In addition, *myc* wild-type and knock-out tumors displayed different transcriptional and metabolic profiles [50].

More recent work further showed the utility of this model. In 2017, the *CTNNB1*/*Yap-1* model was combined with liver-specific *Raptor* knock-out to show a role of mammalian target of rapamycin complex 1 (mTORC1) signaling downstream of YAP-1 in HB [51]. In a second paper from 2017, Yamamoto and colleagues used the hydrodynamic tail vein injection/Sleeping Beauty transposon model to overexpress *myc*, *Yap-1*, *AKT*, *Notch1*, and *Notch2* alone and in combination to generate tumors resembling HB, HCC, and cholangiocarcinoma [52]. Consistent with previous work, the tumors that most closely resembled HB were generated with *Yap-1* and *myc* activation [50]. An interesting study by Wang et al. from 2018 used the *CTNNB1*/*Yap-1* hydrodynamic tail vein injection/Sleeping Beauty transposon model in fumaryl acetoacetate hydrolase (FAH) knock-out animals whose livers had been repopulated by transplant of wild-type, *myc* knock-out, or *chrebp* knock-out hepatocytes [53]. HB tumors originating in *myc*, *chrebp*, or *myc/chrebp* knock-out livers grew more slowly, and clear differences in metabolic and gene expression profiling were shown depending on the genetic backgrounds [53]. In 2019, two different publications explored the tumorigenic capacity of different mutant forms of *CTNNB1* in combination with constitutively active *Yap-1* with this model [54,55]. In a thorough study of the tumorigenic capacity of 14 different mutant forms of *CTNNB1* with *Yap-1*, Zhang and colleagues showed that specific mutant forms of *CTNNB1* determine HB tumor growth rates, survival, histologic features, metabolic features, and transcriptional profiles [55].

The clear benefit of this modeling technique is the ability to study specific effects of genomic aberrations on the development of HB tumors without having to breed a GEM model. As shown by all of this published work, the *CTNNB1*/*Yap-1* model forms a solid foundation on which to study further genes that may contribute to tumorigenesis alongside these constitutively active oncogenes.

## 8. Modeling pediatric HCC and Other Rare Liver Cancers

Unlike the rarity associated with HB, HCC has a long track record of established animal models for study because of the increased incidence of disease. However, to date, most HCC xenograft models that have been developed for further study utilize adult HCC samples [56,57,58]. Along with their generation of HB PDX models, Nicolle and colleagues reported the development of HCC PDX models from pediatric patient tumor samples, but these models were developed from patients that had initially presented with HB and undergone treatment and then experienced recurrence with HCC [23]. Given that HCC is the second most common primary pediatric hepatic malignancy, it is clear that a need exists to develop adequate pediatric HCC models for preclinical studies.

Multiple GEM models for HCC that model hepatic carcinogenesis exist, including a key model that develops with Myc overexpression [33]. In this work, Shachaf and colleagues demonstrated that Myc overexpression specifically in the liver induces HCC development in animals while Myc inactivation results in tumor cells differentiating into hepatocytes and biliary epithelial cells and a rapid loss of AFP [33]. Interestingly, when Myc inactivation was reversed, tumor cells immediately restored their neoplastic features [33]. This work clearly supports a major oncogenic function of Myc in HCC, like what has been shown for HB. This model has even been used in HB research [59].

To be clear, pediatric and adult HCC differ greatly, including in their etiological predisposition and biological behavior, and, therefore, require unique mouse models for meaningful research. HCC in adults usually occurs after chronic necro-inflammation has been ongoing for many years due to alcohol consumption, viral hepatitis, or non-alcoholic fatty liver disease [60]. On the other hand, pediatric HCC develops spontaneously or in the setting of underlying cirrhosis or metabolic, infectious, or vascular liver disease [60]. Notably, survival of children with HCC has improved drastically over the last 30 years, with some patients even responding to chemotherapy while adults with similar severity of disease have worse survival rates [60].

MRT of the liver is an uncommon hepatic malignancy that is very aggressive. While most commonly arising in the kidney, MRT can also occur in the liver. Mortality of MRT of the liver is exceedingly high, approaching 89% [61]. For laboratory studies, only one cell line (G-401) is commercially available, and this cell line has been used to generate subcutaneous and intrarenal mouse xenograft models [62,63,64]. In addition, several subcutaneous PDX mouse models have been generated [65]. In addition, Nicolle and colleagues reported the development of two intrahepatic xenograft models of liver MRT [23].

Angiosarcoma is an undifferentiated sarcoma arising from the endothelial cells and accounts for 1% of all sarcomas [66]. Angiosarcoma of the liver is an extremely rare primary hepatic neoplasm accounting for only 2% of all primary hepatic malignancies with a dismal prognosis of only 3% survival at six years with an average life expectancy of only 6 months after diagnosis [66,67]. Due to the rarity of this disease process, animal models for hepatic angiosarcoma are clearly limited. Dill and colleagues demonstrated that inducible knockout of *Notch1* in mice led to spontaneous hepatic angiosarcoma formation [68]. The authors of this study further demonstrated the ability to generate a cell line from the *Notch1* transgenic model and then use this cell line for further animal modeling with subcutaneous injection [69]. With this second transplanted model, they then showed the efficacy of Sorafenib for this disease [69].

At the time of this review, there has not been a published animal model that accurately recapitulates undifferentiated embryonal sarcoma of the liver (UESL). UESL currently accounts for approximately 10% of all primary liver cancers seen in pediatric patients with an overall survival of greater than 90% at 5 years for patients receiving multimodal therapy regimens, often including orthotopic liver transplantation [70].

Taken together, these studies demonstrate the usefulness of animal models for relevant preclinical work on a range of pediatric hepatocellular malignancies.

## 9. Conclusions

Although pediatric liver cancer is rare, decades of work has resulted in the development of a range of murine models that can be used for diverse preclinical studies (Table 1). Subcutaneous and intrahepatic xenograft models utilizing widely available cell lines or fresh patient samples have been generated and thoroughly validated. Importantly, several techniques have been developed for the generation of these models, including subcutaneous, intrasplenic, and intrahepatic injection of cell suspensions and intrahepatic placement of whole tumor pieces. Subcutaneous tumors tend to be more easily grown and can be clearly monitored for growth and therapy response. Differently, tumors grown in the livers of animals tend to more accurately replicate disease, including the development of a tumor microenvironment, vascularization, and invasive and metastatic disease. For studies of specific genetic aberrations that contribute to tumorigenesis, GEM models have been developed, as well as a novel model that combines a hydrodynamic tail vein injection technique with the Sleeping Beauty-mediated transposon system for liver-specific incorporation of transgenes to manipulate gene expression. Importantly, these models utilize immunocompetent animals, facilitating studies of the immune system in relation to tumors and the tumor microenvironment. The use of GEM models tends to be costly and time-consuming, and such models only replicate the phenotypes associated with the genetic modifications that exist in the animals. The hydrodynamic tail vein injection/Sleeping Beauty transposon model, particularly with coactivation of *CTNNB1* and *Yap-1*, has been used successfully to reveal genes and pathways that contribute to disease, but the tumors generated in animals tend to be initially small, multifocal nodules that eventually replace the entire organ with tumor, which may make quantifying tumor burden difficult. Of note, the intrahepatic models, including the xenograft, GEM, and hydrodynamic tail vein injection/Sleeping Beauty transposon models, can more accurately inform preclinical drug testing because, in the presence of biliary obstruction from a mass effect of tumor within the liver, animals may show toxicities associated with diminished hepatic clearance that they otherwise will not show with tumors grown outside of the liver. Altogether, the use of all of these models already has and will continue to push the field forward with the overall goal of improving outcomes for children that face liver cancer diagnoses.

## Figures and Tables

**Table 1:** Major murine models of HB.

Model	Attributes	Deficits	Primary References
Subcutaneous model	Tumors easily implanted and monitored	Model does not accurately recapitulate tumor microenvironment and vascularization	[15,17]
Splenic injection model	First published model of intrahepatic tumorigenesis	Tumors grow as small, multifocal nodules, which makes quantifying tumor burden difficult	[8,17]
Intrahepatic model	Tumors recapitulate liver microenvironment and show expression of genes and proteins indicative of standard disease	Use of cell lines grown extensively in vitro	[18,19]
Subcutaneous PDX model	Fresh patient samples more closely resemble primary disease	Model does not accurately recapitulate tumor microenvironment and vascularization	[20,21,22,23]
Intrahepatic PDX model	Fresh patient samples closely resemble primary disease and tumors recapitulate liver microenvironment	Limited access to patient samples for model generation	[24]
*LIN28B* GEM model	Use of immunocompetent animals and specific exploration of *LIN28B*	Only models *LIN28B* overexpressing tumors	[25]
*myc*/*CTNNB1* GEM model	Use of immunocompetent animals and specific exploration of *myc* and *CTNNB1*	Less than half of animals develop tumors and most do not survive long after birth for further studies	[26]
*Prom1* Cre-recombination GEM model	Facilitates studies of tumor initiation during development	Not a liver-specific GEM model	[27]
*CTNNB1*/*Yap-1* hydrodynamic tail vein injection/Sleeping Beauty transposon model	Manipulation of genes of interest without the work required for generation of a GEM model	Most animals develop nodules that eventually encompass the entire liver with tumor, which may make quantifying tumor burden difficult	[28]
